# Computational fluid dynamics simulation analysis of the effect of curved rice leaves on the deposition behaviour of droplets

**DOI:** 10.1186/s13007-023-01082-2

**Published:** 2023-10-31

**Authors:** He Zheng, Hao Sun, Yubin Cao, Xiaolan Lv, Chaoxi Wang, Yunfu Chen, Hongfeng Yu, Wei Qiu

**Affiliations:** 1 College of Engineering/Key Laboratory of Intelligent Equipment for Agriculture of Jiangsu Province, Nanjing Agricultural University, Nanjing, 210031 China; 2 Institute of Agricultural Facilities and Equipment, Jiangsu Academy of Agricultural Sciences, Nanjing, 210014 China

**Keywords:** Curved surface structures, Rice leaves, Pesticide droplet, Maximum spreading

## Abstract

**Background:**

Although previous studies on the droplet deposition behaviour of rice leaves have modelled the leaves as flat surface structures, their curved surface structures actually have a significant effect on droplet deposition.

**Results:**

In this paper, the statistical distribution of the coordinate parameters of rice leaves at the elongation stage was determined, computational fluid dynamics (CFD) simulation models of droplet impact on rice leaves with different curvature radii were built, and the effect of leaf curvature radius on the deposition behaviour and spreading diameter of droplets on rice leaves was studied using validated simulation models. The results showed that the average relative errors of the CFD simulation models were in the range of 2.23–9.63%. When the droplets struck the rice leaves at a speed of 4 m/s, the 50 μm droplets did not bounce within the curvature radii of 25–120 cm, the maximum spreading diameters of 200 and 500 μm droplets that just adhered to the leaves were 287 and 772 μm, respectively. The maximum spreading diameters of 50, 200, and 500 μm droplets that just split were 168, 636, and 1411 μm, respectively. As the curvature radii of the leaves increased, the maximum spreading diameter of the droplets gradually decreased, and droplet bouncing was more likely to occur. However, a special case in which no significant change in the maximum spreading diameter arose when 50 μm droplets hit a leaf with a curvature radius exceeding 50 cm.

**Conclusion:**

Splitting generally occurred for large droplets with a small curvature radius and small tilt angle; bouncing generally occurred for large droplets with a large curvature radius and large tilt angle. When the droplet was small, the deposition behaviour was mostly adhesion. The change in spreading diameter after stabilisation was similar to the change in maximum spreading diameter, where the spreading diameter after stabilisation greatly increased after droplet splitting. This paper serves as a reference for the study of pesticide droplet deposition and its application in rice-plant protection.

## Background

Rice diseases, insects, and weeds have been increasing annually in recent years, so there is an urgent need for better disease and pest-prevention and control [1, 2]. Currently, pesticides play an important role in the prevention and control of rice diseases and pests [3, 4]. The use of pesticides can indeed reduce their prevalence and increase crop yield [5, 6], but in the actual application process, only 20–30% of the pesticide is deposited on the rice leaves, and the remaining 70–80% is lost to the soil [7]. Therefore, to improve the deposition of droplets on rice leaves, improve the efficiency of pesticide spraying, and reduce environmental pollution, the factors influencing the impact behaviour of droplets on the leaf surface need to be studied in depth [8, 9].

In recent decades, many scholars have conducted studies on the deposition behaviour of droplets on plant leaves. For example, Xu et al. studied the deposition of pesticide droplets on the surfaces of five different waxy plant leaves and found that the area covered by the droplets was negatively correlated with the content of the leaf’s wax layer [10]. This wax layer imparts hydrophobicity, resulting in droplets bouncing or sliding off the plant-leaf surface. To confront this issue, Damak et al. advanced an intriguing proposition. They suggested that infusing the separation solution with a minimally proportioned, oppositely charged polyelectrolyte, then concurrently atomising this concoction onto the plant foliage using a dual-nozzle apparatus, would trigger an instant precipitation of droplets upon contact. This would in turn generate a hydrophilic surface, effectually securing the droplets to the leaf's exterior and subsequently amplifying their retention capability. However, if two like-charged polyelectrolyte droplets come into contact to form a single large droplet, the precipitation reaction does not occur, similar to the deposition of a single droplet on the leaf surface [11]. In addition, a study found that when individual droplets were deposited on four types of soybean leaf surfaces (distal leaf, proximal leaf, petiole, and basal stem), the distal leaf possessed the largest wetted area of the droplets, followed by the proximal leaf, petiole, and finally the basal stem [12]. By comparing the deposition of droplets at different locations (proximal intervein region, distal intervein region, midvein, and secondary vein) in eucalyptus leaves, Lin et al. found that the wetted area of the droplets on the midvein was the smallest, while that on the secondary vein was slightly larger than that of the intervein [13].

Zhang et al. conducted a qualitative analysis on the influence of the inclination angle of the wet wall surface on the phenomenon of droplet splashing. Their findings revealed that an increase in the tilt angle of the wet wall surface led to an elevation in the critical impact velocity required for the occurrence of the splash phenomenon. [14]. Recently, leaf vibration was reported to affect droplet deposition in air-assisted applications. Compared with static leaf, leaf vibration improves the deposition of low-velocity droplets and small droplets, but this comes with the risk of large-droplet loss [15]. Leaf vibration also increases the spreading diameter of droplets after stabilisation to a small extent [16].

However, all these aforementioned studies consider the leaf to be a flat surface structure. In reality, droplet impact on curved surfaces is a daily phenomenon, such as rain striking canopies and droplets impacting pipe walls in industry [17, 18]. As such, a great deal of research has investigated the effect of surface concavity on droplet-impact spreading on surface structures. Malgarinos et al. pointed out that coating occurs when the initial kinetic energy of the droplet is equal to or higher than the surface energy required to spread the film through the equator of the particle [19]. Furthermore, changes in the Weber number, contact angle, and curvature ratio (the ratio of the initial diameter of the droplet to the diameter of the impacted surface) all have an effect on droplet spreading [20–22]. As the Weber number increases and the contact angle decreases, the maximum spreading factor increases; as the curvature ratio decreases, the film thinning speed decreases, and the remaining thickness increases [21]. When droplets hit the convex and concave surfaces, the spreading factor of the liquid film on the convex surface is greater than that on the concave surface for the same diameter, and this difference decreases as the diameter increases [23]. Thus, a curved surface structure has a significant effect on droplet deposition. Owing to its geometrically continuous curved surface [24], the curvature of different regions of rice leaves varies [25], making it important to study the effect of the curved surface structure on droplet deposition.

However, owing to the complication of small droplets and the accuracy of variable control, it is difficult to test the deposition of droplets on rice leaves. CFD simulation has been demonstrated as an effective means to study droplet impact behaviour on rice leaves [26, 27], which not only saves time and resources but also allows control of the environmental conditions and improves the visibility of microscopic morphological changes of the droplets. To date, most of the CFD simulations of droplet deposition on rice leaves have idealized the leaves as flat surface structures; no relevant studies have simulated droplet deposition on curved surface structures of rice leaves.

In view of this, this study used CFD to simulate the deposition behaviour of droplets colliding with rice leaves in order to construct a theoretical model of droplet deposition behaviour on leaves with different curvature radii. The model performance was compared with the results obtained from actual experiments. The model was also used to investigate the influence of the curved surface structure of the rice-plant leaf on the deposition of droplets and to provide a theoretical basis for improving their deposition in actual application scenarios.

## Materials and methods

### Test platform

**Fig. 1:**
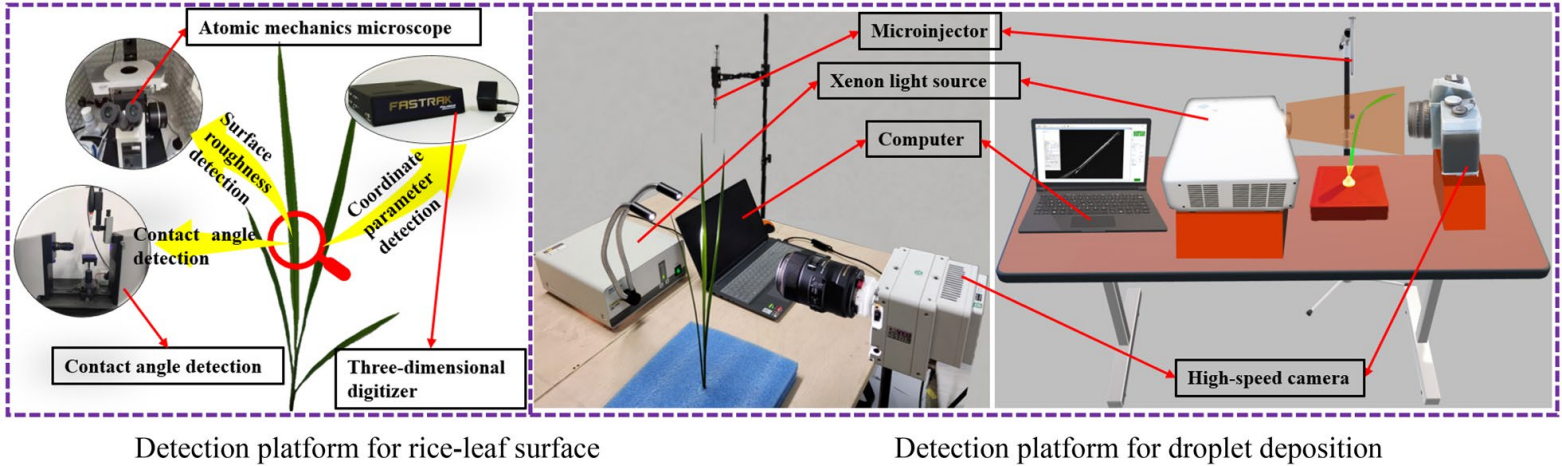
Test platform components

As shown in Fig. 1, the experimental setup consists of a detection platform for the rice-leaf-surface parameters and another for droplet deposition. The rice-leaf surface-parameter detection was divided into three parts: coordinate-parameter detection, contact-angle detection, and surface-roughness detection. The main instruments included three-dimensional digitizer (Polhemus; Fastrak), contact-angle measuring instrument (accuracy ± 0.01°; Biolin; Theta Flow), and atomic force microscope (AFM accuracy ± 0.1 nm; Bruker; Bioscope Resolve). The main instruments comprising the droplet-detection platform included xenon light source (power 100–3000 W; Jingxing Yingchen; HDL), high-speed camera (Full frame shooting rate of 3200 fps; Vision Research; PHANTOM VEO-E310L), microinjector, and computer.

#### Rice-leaf-surface parameters (coordinate parameters, contact angle, and surface roughness)

In this study, Nanjing 46 was selected from the leading agricultural varieties published by the Jiangsu Academy of Agricultural Sciences. This rice variety is widely planted in the southern rice areas of China and has good prospects for promotion. The rice was transplanted on June 5, 2022, and the measurements were recorded on July 12, at the elongation stage. The whole rice plant (Nanjing) was fixed vertically on a fixed base, and the leaves were kept in their natural state. Eight points were marked on each leaf using the leaf pillow as the origin (*x*_*1*_, *y*_*1*_). Three-dimensional digitizer was used to measure the relative coordinates of the marking point (*x*_*i*_, *y*_*i*_) relative to the previous marking point (*x*_*i-1*_, *y*_*i-1*_), and the coordinates were recorded. This procedure was repeated for 200 randomly selected rice samples.

A rice leaf was cut into a 20 mm $\times$ 20 mm sample and affixed to a glass slide, which was set on the sample stage. Next, the microinjector was inserted into the holder to adjust the amount of droplet formed in the needle. Afterwards, the contact-angle measuring instrument was turned on to display the screen; the focus was adjusted so that the droplet was clear. The knob was turned to raise the height of the sample stage so that the surface of the rice leaf contacted the droplet. Then, the sample table was moved down so that the droplet remained on the leaf surface. After the droplet stopped moving, images were recorded to measure the contact angle. This procedure was repeated thrice for each of the 20 randomly selected rice leaves. The average value was the final result of the measurement.

The AFM was powered on, and the computer power supply, chassis low-voltage power supply, high-voltage power supply, and laser power supply were consecutively turned on. The rice leaves were mounted and fixed on the sample stage, and then coarse and fine adjustments were made to complete the probe feeding. The scanning parameters were set. Afterwards, the scanning process began. The appropriate images were stored, and the computer automatically saved their parameters. This procedure was repeated thrice for each of the 20 randomly selected rice leaves. The average value was the final result of the measurement.

#### Detection of droplet deposition

In this test, the backlight method was used to photograph and record, as shown in Fig. 1. The computer was connected to the high-speed camera, which was aligned horizontally along with the xenon light source. The shooting speed was set to 6000 frames per second in order to obtain photos with sufficient image resolution. The rice leaf was fixed on an immobile base and placed in the middle of the camera and the light source; the microinjector was fixed directly above the rice leaf. The liquid sample (water) in the microinjector passed through a stainless-steel injection needle under a certain pressure to produce and deposit individual droplet on the rice leaf. The deposition of the droplets on the rice leaf was recorded using a high-speed camera. Finally, the spreading diameters of the droplets at each moment of the deposition process were obtained. Each experiment was repeated seven times. The maximum and minimum values were removed, and the average of the remaining five spreading diameters was taken as the final result.

### Force process of droplets impacting rice leaves

**Fig. 2:**
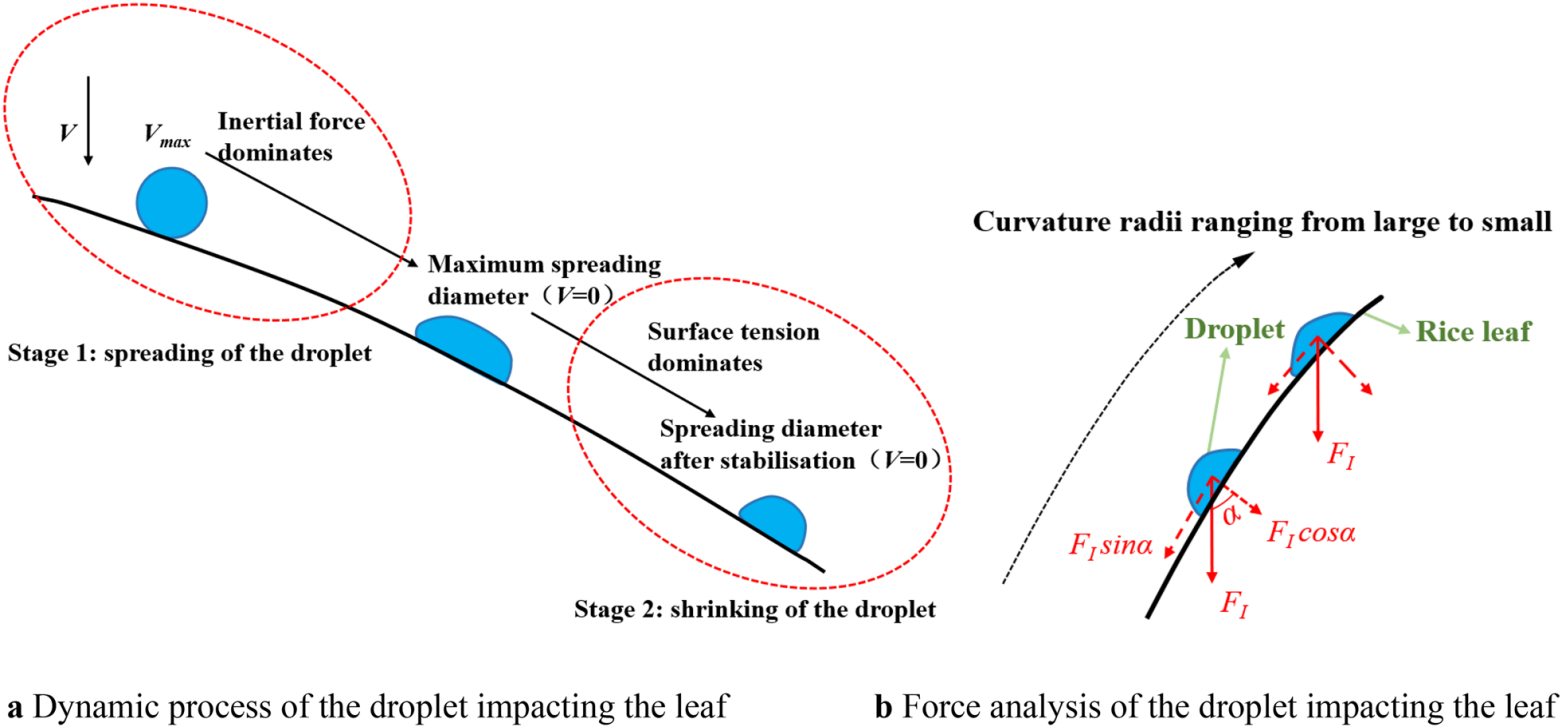
Dynamic process and force analysis of the droplet impacting the rice leaf

As shown in Fig. 2a, the deposition of droplets on rice leaves proceeds in two stages and is subject to the joint action of inertial force, surface tension, and adhesion force [28]. Stage 1: When the droplet just reaches the rice leaves, the droplet velocity is the highest and the inertial force dominates. The droplet then spreads on the leaves. With increasing spreading diameter, the initial kinetic energy is converted into surface energy and dissipative energy. Then, the surface tension gradually increases, slowing the spreading speed of the droplets until it decelerates to 0, when the spreading diameter reaches the maximum. Stage 2: After the droplet spreading diameter reaches the maximum, the surface tension dominates. The surface energy is partly enhanced to shrink the droplet and partly converted to dissipative energy until it reaches a stable state, and the droplet is in the process of shrinking.

When the droplet maximum spreading diameter exceeds a certain threshold value, its deposition behaviour changes. The deposition behaviour is divided into bouncing, adhesion, and splitting. If adhesion and splitting occur, the droplet will eventually be deposited on the leaf, while bouncing will make the droplet fall off the leaf, thus reducing the amount of droplet deposited on the leaf. After the droplet stabilises, the spreading diameter no longer changes. The droplet spreading diameter after stabilisation affects the pesticide absorption rate of the leaf.

Figure 2b shows the force analysis of the droplet impacting the rice leaf. The surface of the rice leaf is convex. The inertial force* F*_*I*_ of the droplet can be divided into the normal inertial force,* F*_*I*_* cosα*, and tangential inertial force, *F*_*I*_* sinα*. The normal inertial force promotes droplet spreading. With the reduction of the curvature radius of the leaf, the normal inertial force gradually increases, the droplet spreading trend is enhanced, and the maximum spreading diameter increases. The smaller the curvature radius, the more convex the leaf surface, the more work is needed to overcome the dissipative energy in the shrinking process, the smaller the surface energy to promote droplet shrinkage, and the larger the spreading diameter after stabilisation.

### Construction of coordinate parameters of rice leaves

**Fig. 3:**
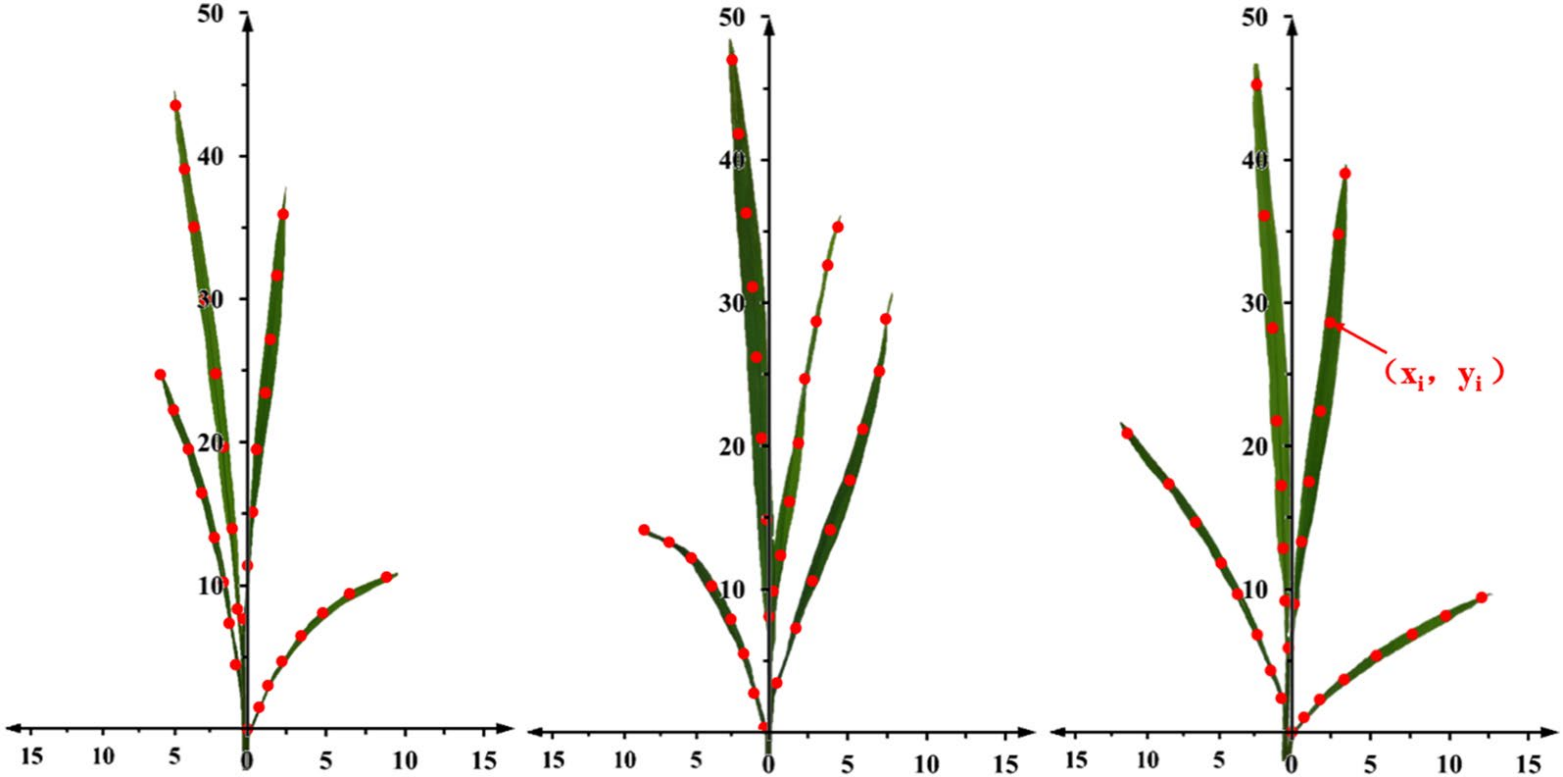
Coordinate point markers of part rice leaves

In order to correctly describe the growth process of rice leaves during bending, this study was conducted in a rice field under normal growth conditions, and the coordinate parameters of the rice leaves were obtained by marking the coordinate points. Each leaf was represented by 1 to 8 points (*x*_*i*_, *y*_*i*_) located on the central vein (see Fig. 3). This method is feasible because in the natural state, rice leaves are generally not twisted and have a smooth curve along the midvein of the leaf in the two-dimensional plane [29]. In addition, this study only addressed the motion state of the droplets and the leaves; it did not consider the interference effects between leaves. Therefore, for statistical purposes, the rice leaves were simplified as shown in Fig. 3, such that all their transverse coordinates were positive and all leaves resided in the first quadrant. Finally, after completing the rice-leaf measurements, the experimental data were fitted to the data generated using Origin 2022 to obtain the coordinate parameters of the rice leaves.

### CFD model construction and validation

#### CFD multiphase and turbulence models

To describe the gas–liquid interface, a volume-of-fluid (VOF) multiphase flow model was used in this simulation. The liquid and gas phases are incompressible Newtonian and isothermal fluids. The governing equations for the VOF model are as follows [30]:

Continuity equation:

1 $$\frac{\partial {\alpha }_{q}{\rho }_{q}}{\partial t}+\nabla \cdot \left({\alpha }_{q}{\rho }_{q}{\overrightarrow{u}}_{q}\right)=0$$

Momentum equation:

2 $$\frac{\partial }{\partial t}\left(\rho \overrightarrow{u}\right)+\nabla \cdot \left(\rho \overrightarrow{u} \overrightarrow{u}\right)=-\nabla \rho +\nabla \cdot \left[\mu \left(\nabla \overrightarrow{u}+\nabla \overrightarrow{{u}^{T}}\right)\right]+\rho \overrightarrow{g}+{\overrightarrow{f}}_{vol}$$

where $\overrightarrow{g}$ is the acceleration of gravity, ${\overrightarrow{f}}_{vol}$ is the equivalent volume force of surface tension, and the subscript *q* denotes the liquid phase L and the gas phase* G*. ${\alpha }_{q}$ is the volume fraction of phase *q*, $\rho$ is the density, and $\mu$ is the kinetic viscosity coefficient, defined as follows:

3 $$\rho =\sum {\alpha }_{q}{\rho }_{q}$$

4 $$\mu =\sum {\alpha }_{q}{\mu }_{q}$$

${\overrightarrow{f}}_{vol}$ is calculated from the continuous surface force (CSF) model, which converts the surface tension into a volume force as the source term of the momentum conservation equation, defined as follows:

5 $${\overrightarrow{f}}_{vol}={\sigma }_{GL}\frac{\rho \kappa \nabla {\alpha }_{q}}{0.5\left({p}_{L}+{\rho }_{G}\right)}$$

In the above equation, ${\sigma }_{GL}$ is the surface tension coefficient, and $\kappa$ is the curvature of the free surface, defined in terms of the dispersion of the surface normal:

6 $$k=\nabla \cdot \overrightarrow{n}, \overrightarrow{n}=\frac{{\alpha }_{q}}{\left|{\alpha }_{q}\right|}$$

The surface normal $\overrightarrow{n}$ is located at the wall cell and is calculated as follows:

7 $$\overrightarrow{n}={\overrightarrow{n}}_{w}\mathit{cos}{\theta }_{d}+{\overrightarrow{t}}_{w}\mathit{sin}{\theta }_{d}$$

where ${\overrightarrow{n}}_{w}$ is the unit vector perpendicular to the wall, ${\overrightarrow{t}}_{w}$ is the unit vector tangent to the wall, and ${\theta }_{d}$ is the contact angle on the wall. In addition, considering the kinetic interface interaction between the rice leaves and the pesticide droplets, a turbulence model was adopted. The corresponding $RNG k-\varepsilon$ equation for the turbulence model is as follows [31]:

8 $$\frac{\partial (\rho k)}{\partial t}+\frac{\partial (\rho k{u}_{i})}{\partial {x}_{i}}=\frac{\partial }{\partial {x}_{i}}\left({\alpha }_{k}{\mu }_{eff}\frac{\partial k}{\partial {x}_{j}}\right)+{G}_{k}+{G}_{b}-\rho \varepsilon -{Y}_{m}+{S}_{k}$$

9 $$\frac{\partial \left(\rho \varepsilon \right)}{\partial t}+\frac{\partial \left(\rho \varepsilon {u}_{i}\right)}{\partial {x}_{i}}=\frac{\partial }{\partial {x}_{i}}\left({\alpha }_{\varepsilon }{\mu }_{eff}\frac{\partial \varepsilon }{\partial {x}_{j}}\right)+{C}_{1\varepsilon }^{*}\frac{\varepsilon }{k}\left({G}_{k}+{C}_{3\varepsilon }{G}_{b}\right)-{C}_{2\varepsilon }\rho \frac{{\varepsilon }^{2}}{k}-{R}_{\varepsilon }+{S}_{\varepsilon }$$

#### CFD model construction

**Fig. 4:**
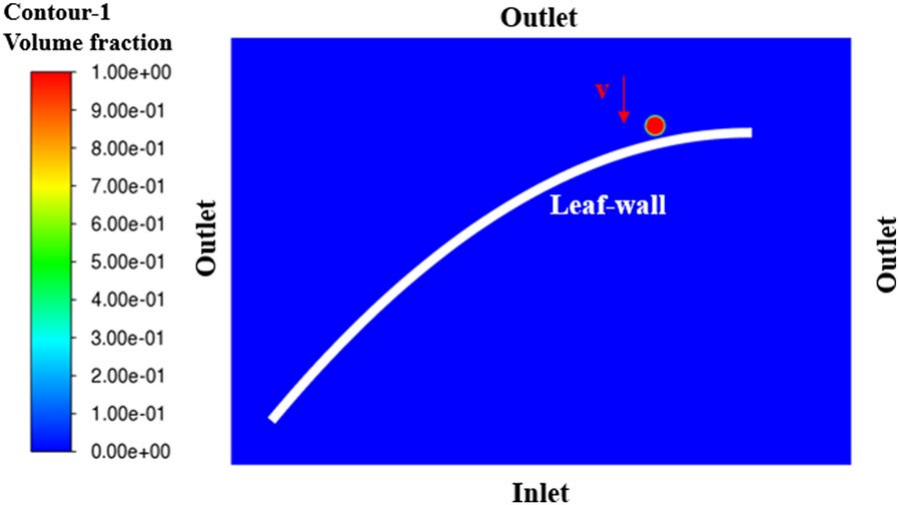
Fluid domain for droplet deposition

**Fig. 5:**
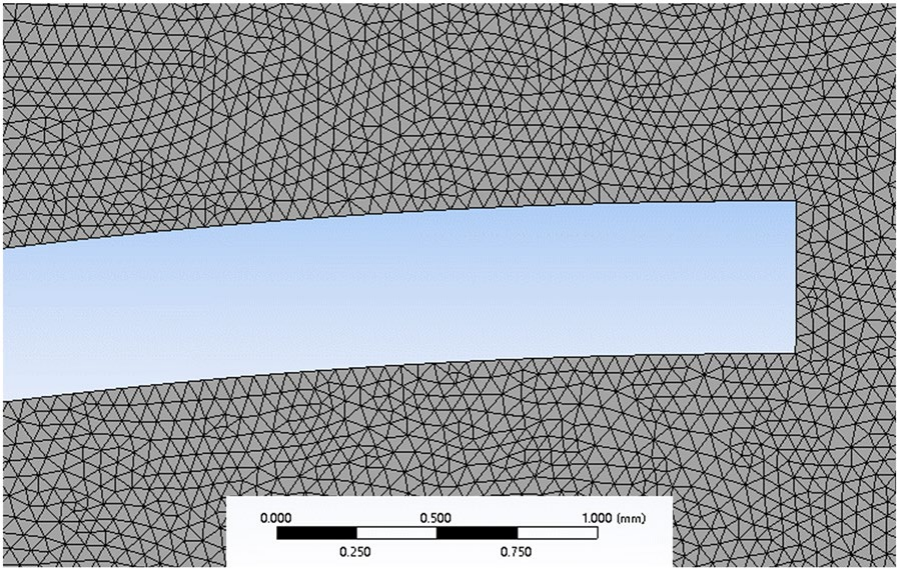
Model meshing

A rice leaf was modelled in a rectangular space, as shown in Fig. 4. The boundary conditions were set as “Inlet” for the bottom boundary of the rectangle, “Outlet” for the remaining three boundaries, and “Leaf-wall” for the rice-leaf boundary. The size of the grid cell was 0.05 mm; the number of nodes was 250,613; and the number of cells was 498,102. The mesh division is shown in Fig. 5.

In the solver, a transient calculation was selected with a gravitational acceleration of 9.81 m/s^2^, and the VOF model and RNG $k-\varepsilon$ turbulence model were adopted. Two phases of air and water were set in the VOF model, with air as the main phase and a surface-tension coefficient of 0.072 N/m for the water–leaf interaction. The corresponding surface roughness and contact angle were set. Then, the standard initialization was selected, and the droplets were generated by customization. The circle was defined internally, and the radius was set separately according to different droplet sizes. After local initialization, the water droplets were customized to contain water in a volume fraction of 1, and the droplets were given a downward initial velocity of 4 m/s.

After completing these operations, the calculation parameters were defined. The calculation was set to compute over an interval of 20 time-steps, where the time-step length was ${10}^{-6}$ s, and the number of time-steps was 16,000. Then, the calculation was initiated, until the residual convergence reached 10^–4^, and the spreading process of the droplets was recorded.

#### CFD model validation

To ensure the feasibility of the model, experiments investigating the droplet impact on rice leaves were conducted. The experimental setup consisted of a droplet-deposition detection platform with a micro-injector to generate droplets of 500 μm and deposit them on the rice leaves with an impact velocity of 4 m/s.

The spray droplet velocity was controlled by changing the height of the spray droplets to the surface of the leaf. The impact velocity was calculated according to the conservation of energy, i.e.:

10 $$mg{h}_{1}=\frac{1}{2}m{{v}_{0}}^{2}$$

11 $${v}_{0}=\sqrt{2g{h}_{1}}$$

where g is the acceleration of gravity, ${h}_{1}$ is the height of the gravity center of the droplets from the horizontal position of the leaves before release, ${v}_{0}$ is impact velocity. The actual velocity of the droplets deviated from the theoretical velocity by approximately 15% under the influence of the environment. The exact velocity of the droplets at the specified elevation was obtained using the data acquisition software connected to the high-speed Phantom cc 3.3 camera. Subsequently, the droplets’ elevation relative to the leaf surface was meticulously calibrated based on the actual velocity data, thereby augmenting the precision of the measured droplet velocities.

Several different rice-leaf tilt angles and curvature radii were selected for the actual tests, and each condition was repeated at least thrice to ensure reproducibility and assess the correlation between the CFD simulation model results and the experimental results.

### Simulation Experiments on the effect of different curvature radii on the deposition behaviour of droplets

The Weber number is an important parameter when a droplet impact on the rice leaves surface, which is calculated by $We=\rho {v}_{0}^{2}{D}_{0}/\sigma$, Among them,* v*_*0*_ is the droplet impact velocity, ${D}_{0}$ is the droplet size, and $\sigma$ is the surface tension. In the actual air-assisted spraying process, the droplet size is generally 40–500 μm [32, 33], and the appropriate velocity of the droplet impacting the leaves is between 1.85 and 6.40 m/s [34]. Therefore, the simulation test was carried out at an impacting velocity of 4 m/s. Droplets with sized of 50, 200, and 500 μm were selected to analyse the effect of particle size on the deposition behaviour, their $We$ are 11.11, 44.44, and 111.11, respectively. Then, in order to investigate the impact of droplet deposition behaviour and the subsequent spreading diameter after stabilization, meticulous consideration was given to the selection of curvature radii and tilt angles, tailored to the precise coordinate parameters of the rice leaves. Furthermore, for the purpose of comparative analysis with the rice leaves, a flat surface structure was also introduced for each tilt angle.

## Results

### Statistical distribution of rice-leaf coordinate parameters

**Fig. 6:**
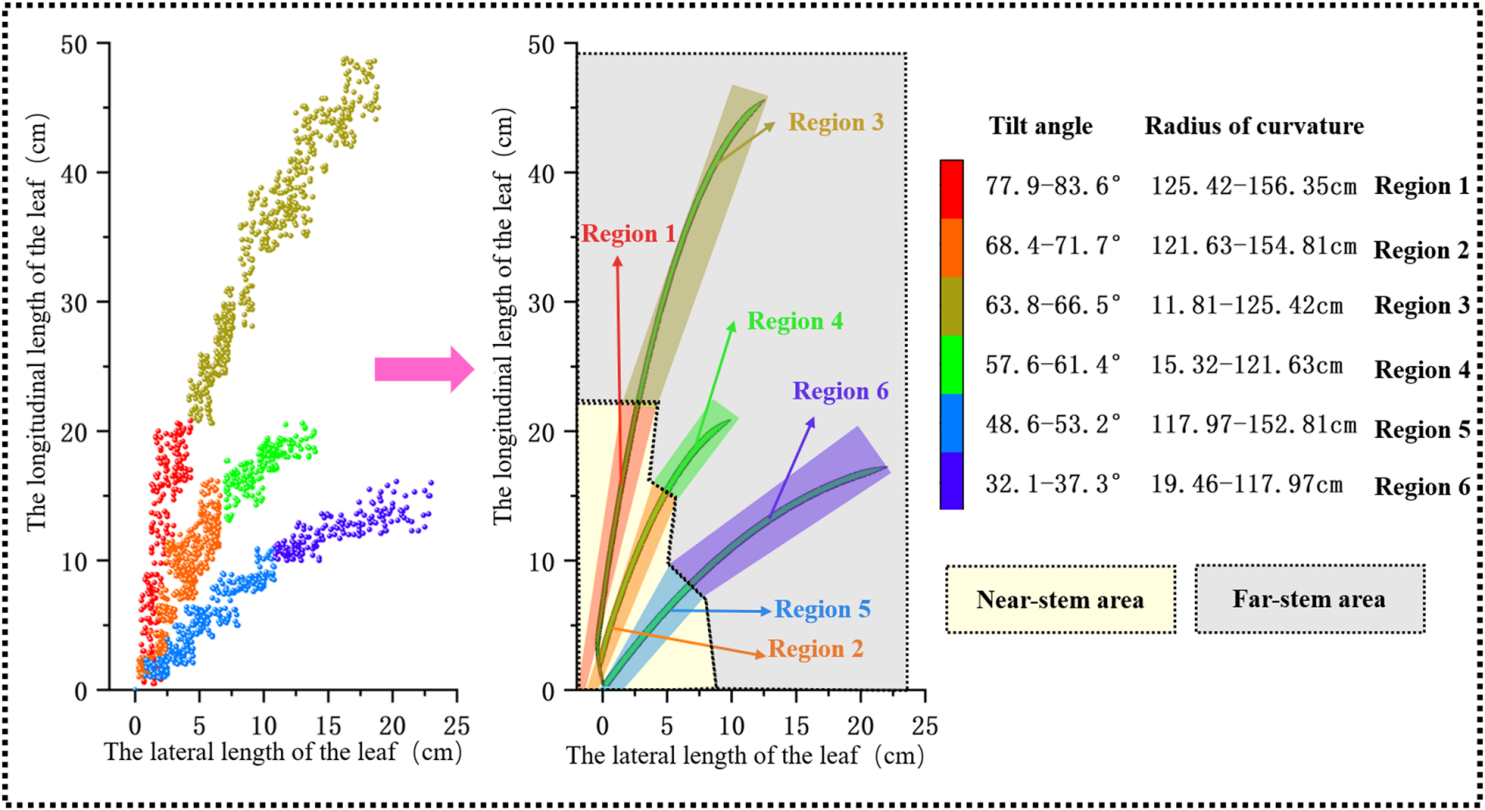
Statistical distribution of coordinate parameters of rice leaves

The coordinate parameters of 200 rice leaves were measured to obtain the statistical distribution (Fig. 6). With respect to the spatial distributions, regions 1, 2 and 5, which are relatively close to the stem, were referred to as the near-stem areas, while regions 3, 4, and 6, which lie farther from the stem, were referred to as far-stem areas.

The range of the leaf tilt angle is 32.1–83.6°, where region 1 has a tilt angle of 77.9–83.6° and a curvature radius of 125.42–156.35 cm; region 2 has a tilt angle of 68.4–71.7° and a curvature radius of 121.63–154.81 cm; region 3 has a tilt angle of 63.8–66.5° and a curvature radius of 11.81–125.42 cm; region 4 has a tilt angle of 57.6–61.4° and a curvature radius of 15.32–121.63 cm; region 5 has a tilt angle of 48.6–53.2° and a curvature radius of 117.92–152.81 cm; and region 6 has a tilt angle and curvature radius of 32.1–37.3° and 19.46–117.97 cm, respectively. In this study, tilt angles of 80°, 70°, 65°, 60°, 50°, and 35° were selected to represent regions 1–6, respectively, in the analysis. In addition, the surface roughness and contact angle of the rice leaves were measured as 1021 nm and 121.3°, respectively.

### CFD model validation

To verify the accuracy of the simulation model, both simulations and experiments were conducted in which 500 μm droplets impacted rice leaves at 4 m/s with a tilt angle of 60° and curvature radii of 25, 50, and 100 cm, and with a tilt angle of 70° and curvature radius of 130 cm.

**Fig. 7:**
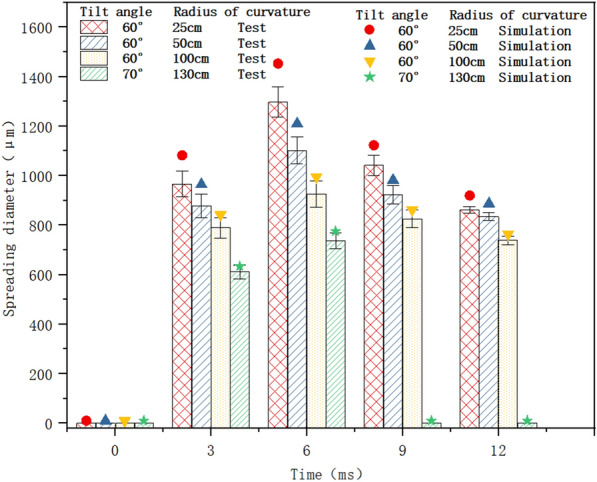
Comparison of simulated and experimental spreading diameters of 500 μm droplets impacting rice leaves at 4 m/s (a spreading diameter of 0 at 0 ms denotes no contact, and a spreading diameter of 0 thereafter indicates bouncing)

**Fig. 8:**
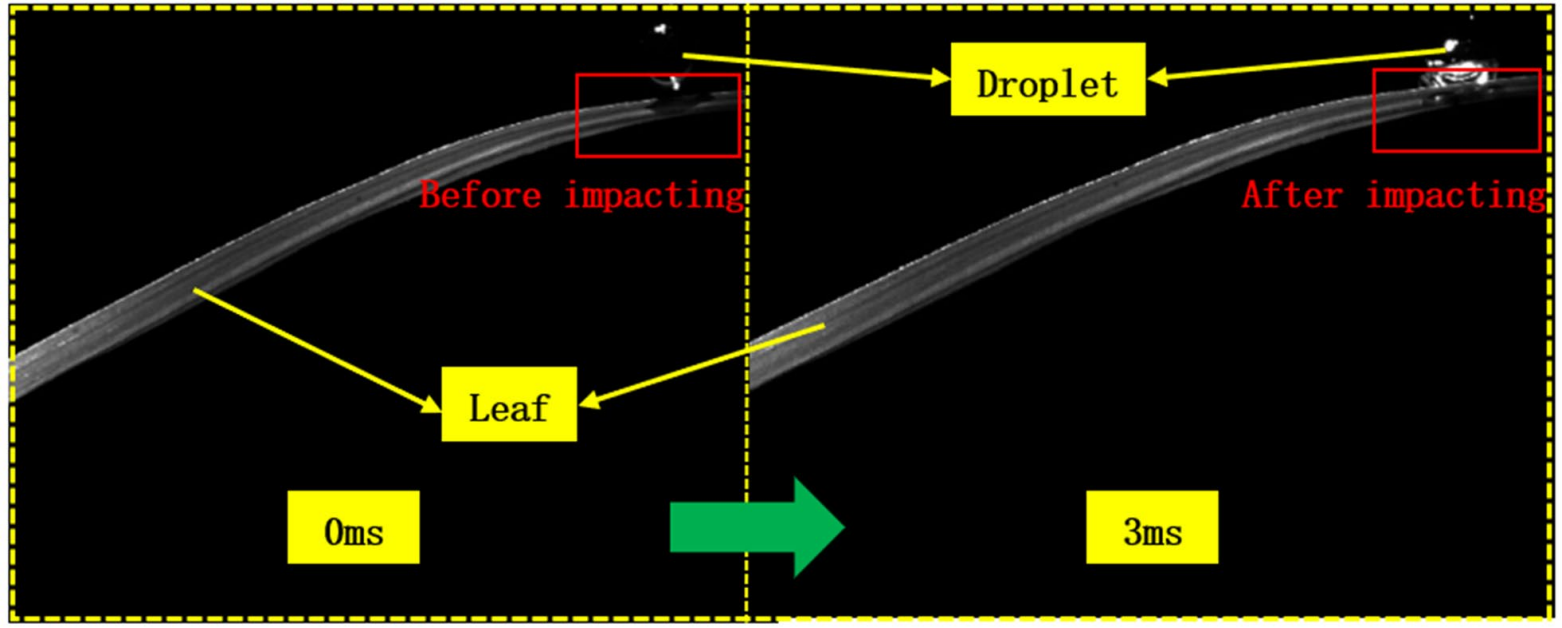
Rice leaves depressed by the impacting droplets

As shown in Fig. 7, the simulated values of the droplet spreading diameters were typically higher than the experimental values under the same conditions. The phenomenon is not accidental but is caused by the downward depression of the droplets impacting the rice leaves in the actual tests (see Fig. 8), which led to slightly larger leaf curvature radii, as listed in Fig. 2b. When the leaf curvature radius increased, the normal inertial force decreased, and the tendency of the droplets to spread declined. Since the deformation caused by the impact is not accounted for in the simulation model, the simulated spreading diameters are slightly higher than the experimental values.

**Fig. 9:**
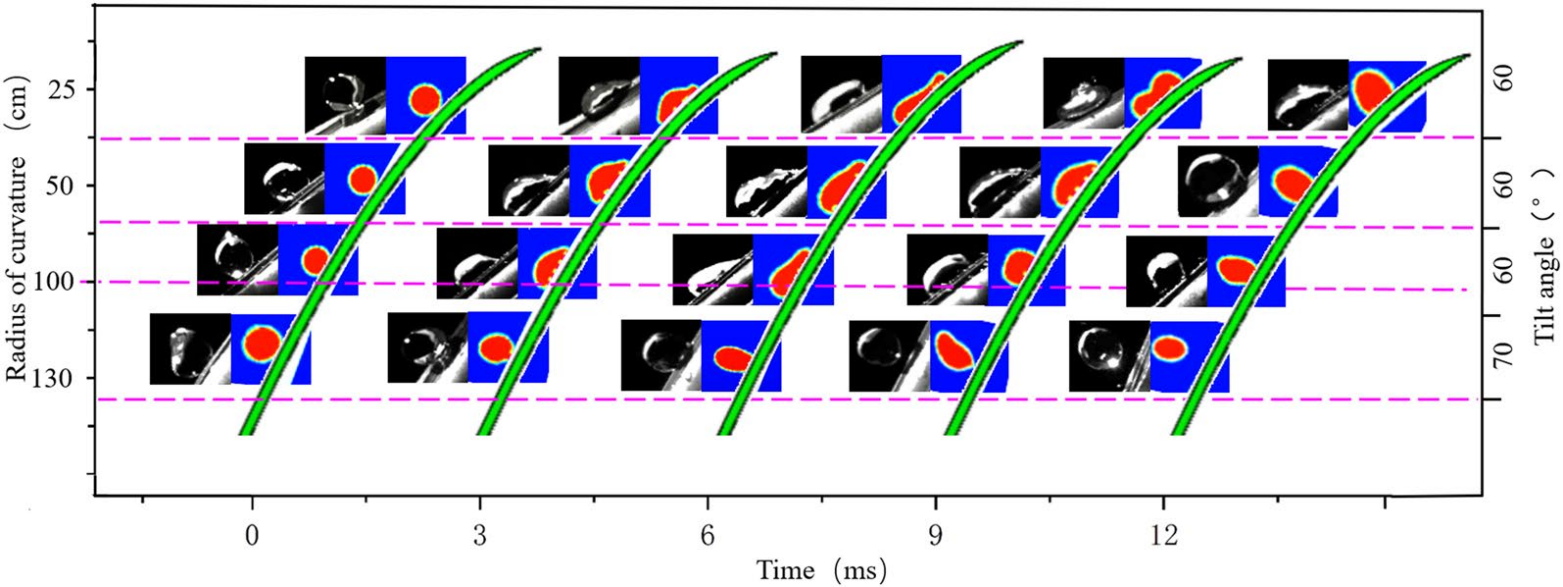
Simulated and experimental dynamic change process of 500 μm droplets impacting rice leaves at 4 m/s

It can also be observed that the curvature radius and time are the main factors affecting the accuracy of the simulation model: the larger the curvature radius, the smaller the average relative error. When the curvature radii of the leaves are 25, 50, 100, and 130 *cm*, the average relative error between the simulation value and test value is 9.63, 8.08, 5.54, and 2.23%, respectively. When the same impact is applied, the rice leaf is closer to the stem, the moment is smaller, and the magnitude of depression is smaller; thus, the spreading diameter of the droplets on the leaf’s surface is reduced to a lesser extent, and the effect of leaf deformation is not considered in the simulation model. At 3 ms and 6 ms, the average relative errors between the simulation value and experimental value are 8.01% and 8.61%, respectively, which are relatively large. At 9 ms and 12 ms, the average relative errors are lower, at 4.70% and 4.08%, respectively. As shown in sect. “Force process of droplets impacting rice leaves”, the deposition process of the droplets is subject to the joint action of inertial force and surface tension. Within 0–6 ms, the droplet spreads and inertial force dominates, but it retracts in the period of 6–12 ms, when surface tension is dominant and inertial force is less influential. Therefore, the normal inertial force decreases, and the droplet-spreading diameter has a greater influence in 0–6 ms and a smaller influence after 6 ms, as shown in Fig. 9. The simulation results of the droplet spreading diameter and profile basically match with the results of the actual test, so the model can be used to determine the influence of the surface structure of rice leaves on droplet deposition behaviour.

### Comparison of the deposition behaviour of droplets on curved and flat surface structures of rice leaves

For the leaves at tilt angles of 35, 60, and 65°, the deposition behaviours of the droplets were observed at curvature radii of 25, 50, and 100 cm, while for tilt angles of 50, 70, and 80°, they were observed at curvature radii of 130, 140, and 150 cm. The deposition behaviours of the droplets on these curved surface structures were compared with those of the droplets on flat surface structures at each tilt angle.

**Fig. 10:**
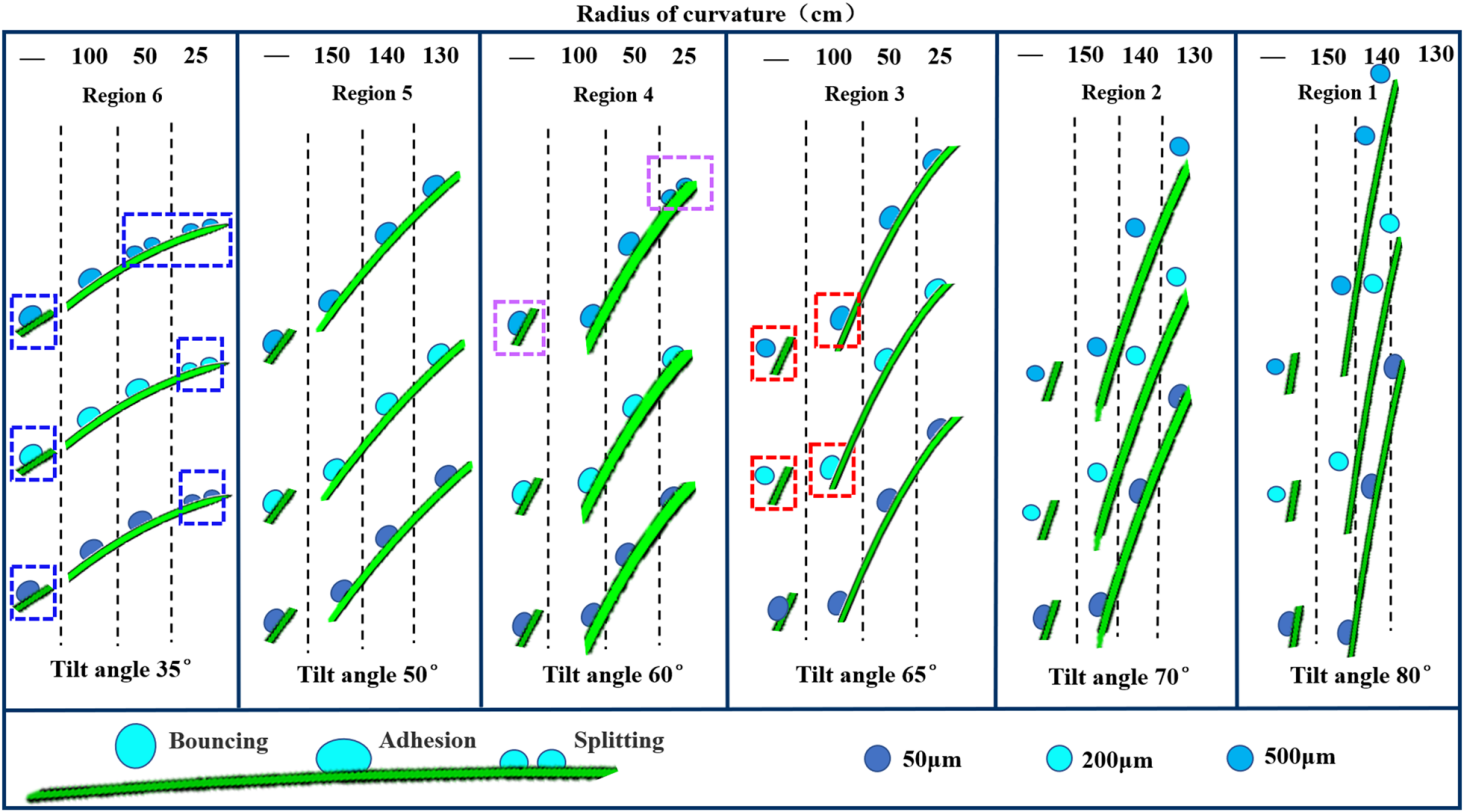
Deposition behaviours of droplets of different particle sizes impacting curved and flat surface structures of rice leaves at 4 m/s (denoted by—, same as below)

Figure 10 shows the deposition behaviours of 50, 200, and 500 μm droplets impacting rice leaves with different tilt angles at a velocity of 4 m/s. It can be seen that the deposition behaviour is inconsistent on curved or flat surface structures in the far-stem region (leaf tilt-angles of 35, 60, and 65°), and droplets are more likely to bounce on flat surface structures. At a tilt angle of 35°, 50 μm droplets tend to split on curved surface structures and adhere to flat surface structures. Droplets with a size of 200 μm adhere to curved surface structures but bounce on flat surface structures when the tilt angle is 65°; when the particle size is increased to 500 μm, splitting occurs on the curved surface structures at tilt angles of 35° and 60°, adhesion occurs on the flat and curved surface structures at 65°, and bouncing occurs on the flat surface structures at 65°. Compared with the flat surface structures, the surface of the curved structure is convex, and the solid–liquid contact area increases rapidly, providing an additional driving force for droplet spreading on the leaf and thus affecting the deposition behaviour of droplets on the leaf. In addition, the deposition behaviour of droplets was found to be consistent on curved and flat surface structures in the near-stem region (leaf angles: 50, 70, and 80°). When the tilt angle is 50°, the droplets adhere to both curved and flat surface structures, and the droplets bounce on both surface structures when the tilt angles are 70° and 80°. This is due to the large curvature radius of the leaf in the near-stem region, which is similar to a flat surface and can thus be equated to a flat surface structure.

### Influence of curved surface structures on the selection of droplet deposition behaviours in rice leaves

**Fig. 11:**
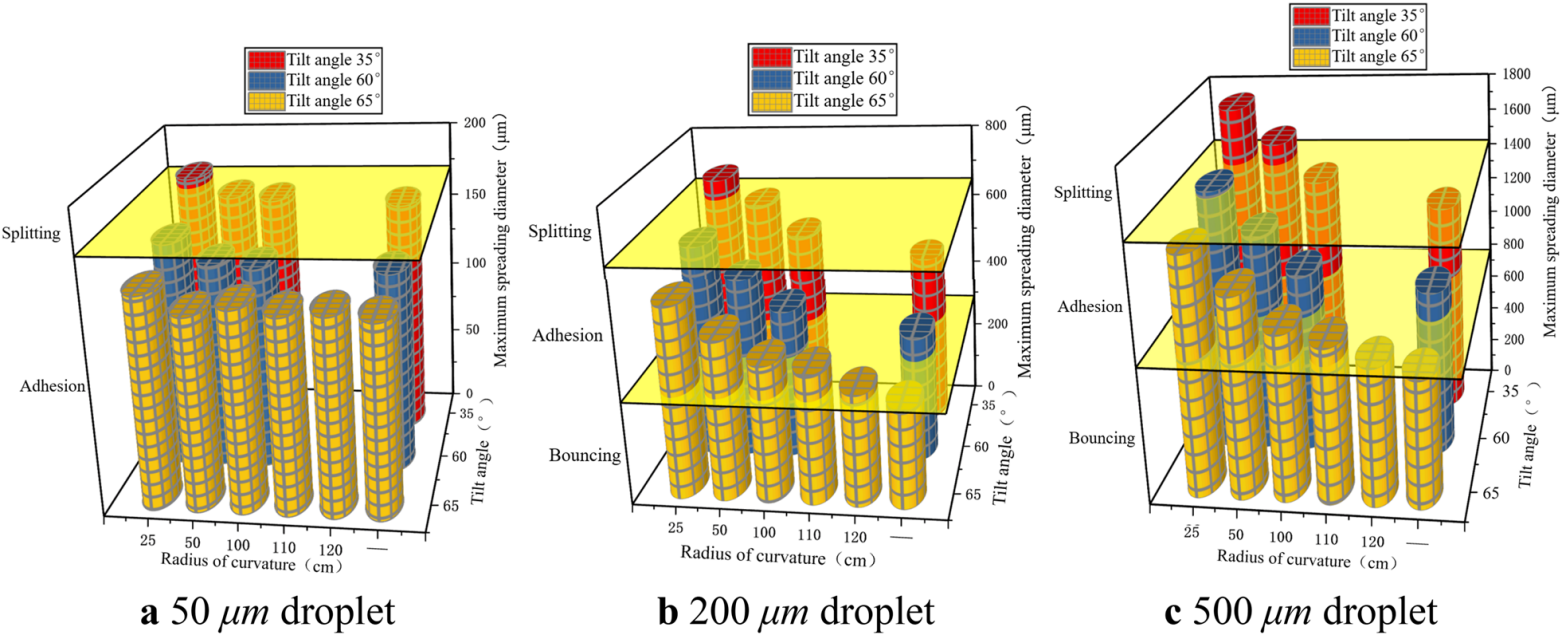
Maximum spreading diameters of droplets deposited on rice leaves upon impacting the leaves at 4 m/s

As shown in Fig. 11, when 50, 200, and 500 μm droplets impact the rice leaves in the far-stem zone at a speed of 4 m/s, the maximum spreading diameter of the droplets decreases significantly with the increase of the curvature radius of the leaves within the curvature radius of 25–120 cm, and the droplets were more likely to bounce. This is because the normal inertial forces of the droplets decrease as the curvature radii of the leaves increase, and the spreading speed of the droplets slows, reducing the maximum spreading diameter (it can be understood that the flat surface structure depicted in Sect. “Comparison of the deposition behaviour of droplets on curved and flat surface structures of rice leaves” is the limit state). However, special cases exist. For example, when 50 μm droplets impact the leaf with a curvature radius of > 50 *cm*, the normal inertia force is significantly reduced owing to the influence of droplet mass and inertial force decomposition, and the maximum spreading diameter remains relatively unchanged and basically stable at ≈ 160 μm (for tilt angles of 35, 60, and 65°). In addition, when the curvature radius is constant, the maximum spreading diameter of the droplets on the leaf decreases with increasing tilt angle and changes significantly at tilt angles of 35, 60, and 65°; the droplets are also more prone to bouncing.

At the same time, Fig. 11 shows that when the droplets impact the rice leaves at 4 m/s, the 50 μm droplets do not bounce, and the maximum spreading diameters of the 200 and 500 μm droplets just after adhesion are 287 and 772 μm, respectively, with corresponding ratios of 143.5% and 154.4% to the initial droplet diameter. In other words, the larger the droplet diameter, the larger is the ratio of the maximum spreading diameter just after adhesion to the initial droplet diameter. The maximum spreading diameters of the 50, 200, and 500 μm droplets just after splitting are 168, 636, and 1411 μm, respectively, and the ratios to the initial droplet diameters are 336.0, 318.0, and 282.2%, respectively, i.e., the larger the droplet diameter, the smaller the ratio of the maximum spreading diameter just after splitting to the initial droplet diameter.

It can be seen that the droplet particle size, curvature radius, and tilt angle have a significant influence on droplet splitting and bouncing. Splitting generally occurs for large droplet sizes, small curvature radii, and small tilt angles; bouncing usually occurs for large droplet sizes, large curvature radii, and large tilt angles; and the deposition behaviour is mostly adhesion when the droplet size is small.

### Effects of curved surface structure on the spreading diameter of droplets after stabilisation in rice leaves

When 50, 200, and 500 μm droplets impacted rice leaves in the far-stem area at a speed of 4 m/s, the variation of the spreading diameter after the droplet stabilises is similar to that of the maximum spreading diameter. As shown in Fig. 2a, the first phase of droplet deposition is spreading, and the second phase is contraction. As the leaf curvature radius decreases, the leaf surface becomes more convex, requiring more work to offset the dissipative energy during the contraction process and less surface energy to promote droplet contraction. As a result, the post-stabilisation spreading diameter increases.

**Fig. 12:**
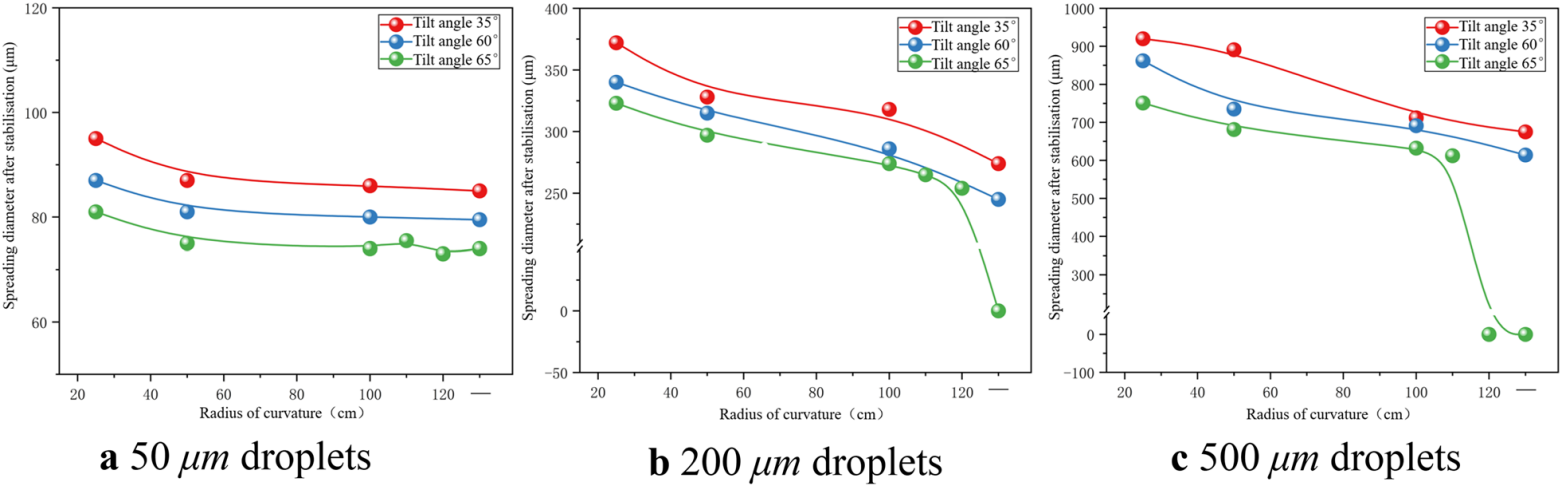
Spreading diameters of droplets after deposition stabilises on rice leaves upon impacting the leaves at 4 m/s

As can be seen from Fig. 12, the stabilised spreading diameters of the 50, 200, and 500 μm droplets after adhesion range from 73 to 89, 248–341, and 612–748 μm, respectively, corresponding to increases of 46.0–78.0%, 24.0–70.5%, and 22.4–49.6% compared with the initial particle sizes. The 50 and 200 μm droplets split only once in the curvature radius range of 25–120 cm, and the stabilised spreading diameters were 95 and 371 μm, respectively, while the spreading diameters of the 500 μm droplets after stabilisation were 862–918 μm, representing increases of 90, 85.5, and 72.4–83.6% compared with the initial particle sizes of 50, 200, and 500 μm, respectively. It is evident that when the droplets split, the droplet spreading thickness on the leaf will be reduced, while the volume after splitting remains unchanged, and the spreading diameter after stabilisation is greatly increased, which is beneficial to the application process. The spreading diameter after stabilisation plummeted to 0 after the droplet bounced, so the ideal situation during the application process is to realize splitting as well as prevent the droplet from bouncing.

## Discussion

In rice pest and disease control, pesticides are mainly applied by air-assisted spraying on the stems and leaves. However, droplets cannot be effectively deposited on the surface of rice leaves owing to phenomena such as bouncing, splashing, and rolling off [35]. As such, many researchers have investigated the microscopic dynamics of spray droplets and predicted droplet deposition behaviour by impacting the droplets on target leaf models [36–38], but rice leaves have a complex curved structure in reality, and most of the previous studies did not consider the influence of the curved surface structure on the droplet deposition behaviour. Therefore, the study of spray droplets impacting leaves with curved surface structures has a significant impact on the study of pesticide deposition characteristics, thus the improvement of pest and disease control technology.

In this paper, a model simulating spray droplets impacting rice leaves with structures of different curvature radii was developed to study the deposition behaviour of droplets upon impact. The results show that when the droplet particle sizes were 200 and 500 μm, the maximum spreading diameters of the droplets on the rice leaves in the far-stem region gradually decreased with the increase in leaf curvature radius, making the droplet more likely to bounce. By contrast, the maximum spreading diameters of the droplets did not obviously change when 50 μm droplets impacted leaves possessing larger curvature radii. It was found that the maximum spreading diameters of the droplets decrease with increasing curvature radius of the curved surface when the droplets impact the surface, but this behaviour occurs within a certain range of droplet particle sizes. Liu et al. carried out theoretical and experimental analyses of the droplet impact behaviour on curved surfaces and found that the maximum spreading diameters of the droplets decrease with increasing curvature radii of the surfaces for droplets with a size of 3.3 mm. [23] In addition, Usawa et al. developed a numerical model for droplets impacting spherical surfaces by controlling the impact of 4.8 mm droplets on surface structures with different curvature radii. They found that the maximum spreading diameters of the droplets decrease when the surface curvature radius is larger [39], with the droplet characteristics and contact angles also affecting the droplet deposition behaviour [40, 41].

In addition, different leaf tilt angles also lead to different deposition behaviours of the spray droplets, where an appropriate reduction of the tilt angle is conducive to increasing the deposition volume [42]. In this study, when the curvature radius was constant, the maximum spreading diameters of the droplets decreased gradually with increasing tilt angles of 35, 60, and 65°, and the droplets were more prone to bouncing. However, this phenomenon was not obvious for small droplets, which generally exhibited adhesion. A reduction in droplet diameter can inhibit droplet bouncing [14], but exceedingly small droplets appear to volatilize before depositing on the target; therefore, attaining reasonable control of the droplet size in practical applications is a problem to be solved.

The CFD simulation model established in this paper proves that the curved structure of rice leaves influences the deposition behaviour of spray droplets, but the following limitations still exist. ① When the droplets impact the rice leaves, the droplets will depress the rice leaves downwards owing to its own impulse, but the simulation model does not consider this factor, which will be included in subsequent research. ② In this paper, water was used in place of pesticides with complex ingredients, hence the simulation results may deviate from those obtained in an actual application scenario. These issues will be investigated in subsequent studies to further improve the simulation accuracy.

## Conclusions

The deposition behaviours of spray droplets upon impacting rice leaves with different curvature radii were investigated using CFD simulations combined with the statistical distribution of the coordinate parameters of the rice leaves. The dynamic process of droplet deposition was elucidated through simulation tests, and actual experiments were conducted to verify the accuracy of the simulation results. The tests revealed that the curvature radii of rice leaves range from 15.32 to 156.35 cm, and the tilt angles range from 32.1 to 83.6°. The corresponding curvature radii for tilt angles of 48.6–53.2°, 68.4–71.7°, and 77.9–83.6° were in the ranges of 117.92–152.81 cm, 121.63–154.81 cm, and 125.42–156.35 cm in the near-stem area, i.e., the region close to the stem. The tilt angles in the far-stem zone range from 32.1 to 83.6°. The curvature radii corresponding to tilt angles of 32.1–37.3°, 57.6–61.4°, and 63.8–66.5° were in the range of 19.46–117.97 cm, 15.32–121.63 cm, and 11.81–125.42 cm, respectively.

The deposition behaviours of 50, 200, and 500 μm droplets impacting rice leaves at 4 m/s were consistent for curved and flat surface structures in the near-stem area, while they differed in the far-stem area. Upon impacting the leaves in the far-stem area, the 50 μm droplets did not bounce, while the 200 and 500 μm droplets that just adhered to the leaves with maximum spreading diameters of 287 and 772 μm, respectively. The maximum spreading diameters of the 50, 200, and 500 μm droplets immediately after just splitting were 168, 636, and 1411 μm, respectively. As the curvature radii of the leaves increased, the maximum spreading diameters of the droplets gradually decreased, and the droplets were more prone to bouncing (the flat surface structure can be considered the limit state). However, one special case in which no significant change in the maximum spreading diameter occurred when 50 μm droplets impacted leaves with curvature radii of > 50 cm. When the curvature radius remained the same, the maximum spreading diameter of the mist droplets gradually decreased as the tilt angle increased in the order of 35, 60, and 65°, and bouncing was more likely to happen. In general, splitting occurred for large droplet sizes, small curvature radii, and small tilt angles; bouncing occurred for large droplet sizes, large curvature radii, and large tilt angles; and adhesion occurred for small droplet sizes.

The variation in the post-stabilisation spreading diameter was similar to that in the maximum spreading diameter when the droplets impacted the rice leaves at 4 m/s. After the droplets split, the post-stabilisation spreading diameter increased substantially.

This study investigated the impact kinetics of spray droplets on the curved surface structure of rice leaves and elucidated the influence of the leaf curvature radius on droplet deposition. Therefore, our study can serve as a reference for other studies on pesticide droplet deposition and disease and pest control technology.

## Data Availability

The datasets used and/or analyzed during the current study are available from the corresponding authors upon reasonable request.

## List of Symbols

${\overrightarrow{f}}_{vol}$: Equivalent volume force of surface tension
${\overrightarrow{n}}_{w}$: Unit vector perpendicular to the wall
${\overrightarrow{t}}_{w}$: Unit vector tangent to the wall
$${h}_{1}$$: Height
$\overrightarrow{g}$: Acceleration of gravity
$$\overrightarrow{n}$$: Surface normal
$${v}_{0}$$: Impact velocity
F_I_: Normal inertial force
m: Meters
cm: Centimeter
nm: Nanometer
μm: Micron
*q*: Phase
s: Second
*V*: Droplet velocity
*V*_*max*_: Maximum droplet velocity
*We*: Weber number

## Greek symbols

*α*: Impact angle between droplets and rice leaves
$\kappa$: Curvature of the free surface
$\mu$: Kinetic viscosity coefficient
$$\rho$$: Density
${\sigma }_{GL}$: Surface tension coefficient
${\alpha }_{q}$: Volume fraction of phase
${\theta }_{d}$: Contact angle on the wall

## References

[1] Cheng L, Huang FG, Jiang Z, Lu BY, Zhong XH, Qiu YF (2021). Improved phenotyping procedure for evaluating resistance in rice against gall midge (*Orseolia oryzae*, Wood-Mason). Plant Methods.

[2] Tudi M, Daniel Ruan H, Wang L, Lyu J, Sadler R, Connell D (2021). Agriculture development, pesticide application and its impact on the environment. Int J Environ Res Public Health.

[3] Fang C, Xu Y, Ji Y (2022). Part-time farming, diseases and pest control delay and its external influence on pesticide use in China’s rice production. Front Environ Sci.

[4] Dunn L, Latty T, Van Ogtrop FF, Tan DK (2023). Cambodian rice farmers’ knowledge, attitudes, and practices (KAPs) regarding insect pest management and pesticide use. Int J Agric Sustain.

[5] Perthame L, Petit S, Colbach N (2023). Modelling weed seed predation by carabids and its effects on crop production under contrasted farming systems. Eur J Agron.

[6] Menesch J, Godde C, Venables W, Renard D, Richardson A, Cobelli O (2023). Agricultural diversification for crop yield stability: a smallholder adaptation strategy to climate variability in Ethiopia. Reg Environ Change.

[7] Wang SJ, Wang HJ, Li T, Li C, Zhong XM, Zhou YJ (2016). Wetting property representation of pesticides on the crop leaf surfaces. Bangladesh J Bot.

[8] Bao Z, Wu Y, Liu R, Zhang S, Chen Y, Wu T (2023). Molecular selection and environmental evaluation of eco-friendly surfactants to efficiently reduce pesticide pollution. J Clean Prod.

[9] Jiang Y, Yang Z, Xu X, Shen D, Jiang T, Xie B (2023). Wetting and deposition characteristics of air-assisted spray droplet on large broad-leaved crop canopy. Front Plant Sci.

[10] Xu LY, Zhu HP, Ozkan HE, Bagley WE, Krause CR (2011). Droplet evaporation and spread on waxy and hairy leaves associated with type and concentration of adjuvants. Pest Manage Sci.

[11] Damak M, Mahmoudi SR, Hyder MN, Varanasi KK (2016). Enhancing droplet deposition through in-situ precipitation. Nat Commun.

[12] Gimenes MJ, Zhu H, Raetano CG, Oliveira RB (2013). Dispersion and evaporation of droplets amended with adjuvants on soybeans. Crop Prot.

[13] Lin H, Zhou HP, Xu LY, Zhu HP, Huang HH (2016). Effect of surfactant concentration on the spreading properties of pesticide droplets on *Eucalyptus* leaves. Biosyst Eng.

[14] Zhang F, Li JX, Liu QF, Bo HJ (2018). Experiment study of droplet impacting on wetted surface. At Energy Sci Technol.

[15] Cao YB, Xi T, Xu LJ, Qiu W, Guo HB, Lv XL (2022). Computational fluid dynamics simulation experimental verification and analysis of droplets deposition behaviour on vibrating pear leaves. Plant Methods.

[16] Qiu W, Guo HB, Zheng H, Cao YB, Lv XL, Fang J (2022). CFD modelling to analyze the droplets deposition behavior on vibrating rice leaves. Comput Electron Agric.

[17] Deng T, Varanasi KK, Hsu M, Bhate N, Keimel C, Stein J (2009). Nonwetting of impinging droplets on textured surfaces. Appl Phys Lett.

[18] Liu HF, Ma SY, Zhang Z, Zheng ZQ, Yao MF (2015). Study of the control strategies on soot reduction under early-injection conditions on a diesel engine. Fuel.

[19] Malgarinos I, Nikolopoulos N, Gavaises M (2016). A numerical study on droplet-particle collision dynamics. Int J Heat Fluid Flow.

[20] Sahoo PC, Senapati JR, Rana BK (2022). Computational and analytical investigation of droplet impingement and spreading dynamics around the right circular cone. Langmuir.

[21] Sheykhian MK, Kayhani MH, Norouzi M, Kim M, Kim KC (2023). An experimental study on the impact of Boger and Newtonian droplets on spherical surfaces. Phys Fluids.

[22] Fan Z, Liu D, Pan S, Ma J, Chen X (2023). Spreading dynamics of the viscous droplet impacting on a spherical particle. Phys Fluids.

[23] Khurana G. Droplet Impact Hydrodynamics on Curved Surfaces. 2022.

[24] Zhang YH, Tang L, Liu XJ, Liu LL, Cao WX, Zhu Y (2017). Modeling curve dynamics and spatial geometry characteristics of rice leaves. J Integr Agric.

[25] Wang B, Liu Y, Sheng QH, Li J, Tao JH, Yan ZJ (2022). Rice phenology retrieval based on growth curve simulation and multi-temporal sentinel-1 data. Sustainability.

[26] Fan GJ, Wang SY, Bai P, Wang DW, Shi WJ, Niu CQ (2022). Research on droplets deposition characteristics of anti-drift spray device with multi-airflow synergy based on CFD simulation. Appl Sci-Basel.

[27] Li HZ, Zhu H, Jiang ZH, Lan YB (2022). Performance characterization on downwash flow and spray drift of multirotor unmanned agricultural aircraft system based on CFD. Int J Agric Biol Eng.

[28] Labergue A, Gradeck M, Lemoine F (2016). Experimental investigation of spray impingement hydrodynamic on a hot surface at high flow rates using phase Doppler analysis and infrared thermography. Int J Heat Mass Transf.

[29] Shi CL, Zhu Y, Cao WX (2006). A quantitative analysis on leaf curvature characteristics in rice. Acta Agron Sin.

[30] Liu ZH, Li YB, Su MJ, Luo Y, Chu GW (2022). Dispersion phenomena of liquid droplet impacting on the single fiber with different wettabilities. Chem Eng Sci.

[31] Bahramian A, Mohammadi M, Ahmadi G (2022). Effect of indoor temperature on the velocity fields and airborne transmission of sneeze droplets: an experimental study and transient CFD modeling. Sci Total Environ.

[32] Yang XW, Dai ML, Song JL, Zhao JK, He XK (2013). Effect of droplet size, leaf characteristics and angle on pesticide deposition. Trans Chin Soc Agric Eng.

[33] Wang JX, Qi LJ, Xia QJ (2015). CFD simulation and validation of trajectory and deposition behavior of droplets around target affected by air flow field in greenhouse. Trans Chin Soc Agric Eng.

[34] Jia W, Zhu H (2015). Dynamics of water droplet impact and spread on soybean leaves. Trans ASABE.

[35] Xu MH, Li XR, Riseman A, Frostad JM (2021). Quantifying the effect of extensional rheology on the retention of agricultural sprays. Phys Fluids.

[36] Kim HY, Park SY, Min K (2003). Imaging the high-speed impact of microdrop on solid surface. Rev Sci Instrum.

[37] Safavi M, Nourazar SS (2021). Droplet capture with a wetted fiber. Theor Comput Fluid Dyn.

[38] Si ZB, Shimasaki N, Nishida K, Ogata Y, Guo M, Tang CL (2018). Experimental study on impingement spray and near-field spray characteristics under high-pressure cross-flow conditions. Fuel.

[39] Usawa M, Fujita Y, Tagawa Y, Riboux G, Gordillo JM (2021). Large impact velocities suppress the splashing of micron-sized droplets. Phys Rev Fluids.

[40] Kietzig AM (2011). Comments on “An essay on contact angle measurements”—an illustration of the respective influence of droplet deposition and measurement parameters. Plasma Process Polym.

[41] Yan-Peng L, Huan-Ran W (2011). Three-dimensional direct simulation of a droplet impacting onto a solid sphere with low-impact energy. Can J Chem Eng.

[42] Yao W, Wang X, Lan Y, Jin J (2018). Effect of UAV prewetting application during the flowering period of cotton on pesticide droplet deposition. Front Agric Sci Eng.

